# Paraneoplastic Autoimmune Neurological Syndromes and the Role of Immune Checkpoint Inhibitors

**DOI:** 10.1007/s13311-022-01184-0

**Published:** 2022-01-18

**Authors:** Sophie L. Duong, Harald Prüss

**Affiliations:** 1 Department of Neurology and Experimental Neurology, Charité Universitätsmedizin Berlin, Corporate Member of Freie Universität Berlin, Humboldt-Universität Berlin, and Berlin Institute of Health, Charitéplatz 1, 10117 Berlin, Germany; 2 German Center for Neurodegenerative Diseases (DZNE) Berlin, 10117 Berlin, Germany

**Keywords:** Paraneoplastic neurological syndromes, Immune checkpoint inhibitors, Neurological adverse events, Autoantibodies, Novel immunotherapies, Biologicals

## Abstract

The introduction of immune checkpoint inhibitors (ICIs) in oncologic therapies has led to a paradigm shift in cancer treatment. ICIs have increased the overall survival in patients with malignant melanoma, small-cell lung cancer, and many other tumor entities. Despite their clinical benefits, these novel cancer immunotherapies can induce neurological immune-related adverse events (irAEs). Such immune-mediated complications can manifest within the spectrum of paraneoplastic neurological syndromes (PNSs). PNSs are rare immune-mediated complications of systemic cancers that can involve every aspect of the nervous system. The emergence of PNSs with ICI treatment opens further pathways to study the complex immunopathological interplay of cancer immunity, cross-reactive neurological autoimmune phenomena, and effects of ICIs on the immune system. ICI-induced PNSs comprise a diverse antibody repertoire and phenotypic spectrum with severe and life-threatening disease progression in some cases. Timely diagnosis and urgent interventions are pivotal for a favorable neurologic and oncologic outcome. This review focuses on the pathogenesis of cancer immunotherapy and the disruption of immune tolerance in PNSs and provides an overview of the most pertinent clinical manifestations and principles of diagnostic and therapeutic managements in light of the expected increase in PNSs due to the widespread use of ICIs in clinical practice. This review further discusses potential and evolving concepts of therapeutic monoclonal antibodies for the treatment of PNSs.

**Supplementary Information:**

The online version contains supplementary material available at 10.1007/s13311-022-01184-0.

## Introduction

The adoption of novel cancer immunotherapies in general and notably the rise of ICIs in particular transform the oncologic therapeutic landscape [1]. Recent years have shown that ICIs improve patient survival outcomes and achieve long-term remissions in multiple advanced malignancies such as metastatic melanoma, small-cell lung cancer (SCLC), non-small-cell lung cancer (NSCLC), and Hodgkin lymphoma, among others [1–3]. ICIs constitute monoclonal antibodies that block negative regulators of T cell activation, thus promoting T cell-mediated antitumor immune responses to overcome the evasive immune mechanisms of cancer cells [4]. Targets for the therapeutic blockade include the inhibitory immune checkpoints cytotoxic T lymphocyte-associated antigen 4 (CTLA-4) and the programmed cell death protein 1 receptor (PD-1) and its ligand PD-L1 [4].

The clinical benefits of ICI therapy have increased the risk for severe immune-related adverse events (irAEs), resulting from the broad enhancement of endogenous immune responses. ICI-induced irAEs can affect any organ system, including the nervous system [5–7]. Neurological irAEs are rare complications with an estimated overall incidence of 3.8% with anti-CTLA-4 therapy, 6.1% with anti-PD-1 therapy, and 12% with a combination of both. Severe neurotoxicities occur in less than 1.0% of cases [8]. Yet, neurological irAEs are clinically relevant as long-term sequelae remain in 40–60% of patients and 6–15% of all neurological toxicities are fatal [9–11].

Neurological symptoms usually develop within 3 months of ICI treatment [5, 6, 12]. The clinical features of neurological irAEs can be diverse with multifocal involvement, affecting any part of the nervous system. A subset of ICI-related neurotoxicities presents as PNSs. These neurotoxicities are of particular clinical concern for their often strikingly rapid clinical deterioration and severe, life-threatening manifestations that are associated with a poor neurological outcome if left untreated [12–14]. Irrespective of ICI treatment, classical PNSs are defined as immune-mediated neurological disorders that can affect any part of the nervous system and demonstrate a tight association with cancer [15]. The widespread use of ICIs in oncology, especially in cancers known for their paraneoplastic association (such as SCLC), is predicted to increase the incidence of PNSs [3, 16, 17]. The occurrence of these disorders within the context of such immunotherapies offers new perspectives on studying the immunological mechanisms underlying tumor immune surveillance and the collapse of immune tolerance resulting in PNSs. When PNSs arise as irAEs, it is important to exclude alternative diagnoses to pave the way for further management. The recognition of stereotyped neurological phenotypes, the detection of neuronal autoantibody biomarkers, and specific neuroimaging abnormalities are the pillars of establishing the diagnosis.

This review provides an overview of distinct clinical features of PNSs in the framework of ICI treatment and diagnostic approaches with focus on neuronal antibody association. This article further addresses proposed immunopathogenic principles of ICIs as triggers of PNSs, and the derived therapeutic strategies that are most pertinent to the treating neurologist. In view of the expanding indications of ICIs in oncology and the anticipated increased PNSs’ prevalence, this review aims to raise awareness among treating clinicians to timely identify these disorders, because if left untreated, PNSs are associated with high morbidity and mortality.

## Cancer Immunity and Immunopathology of PNSs

**Fig. 1:**
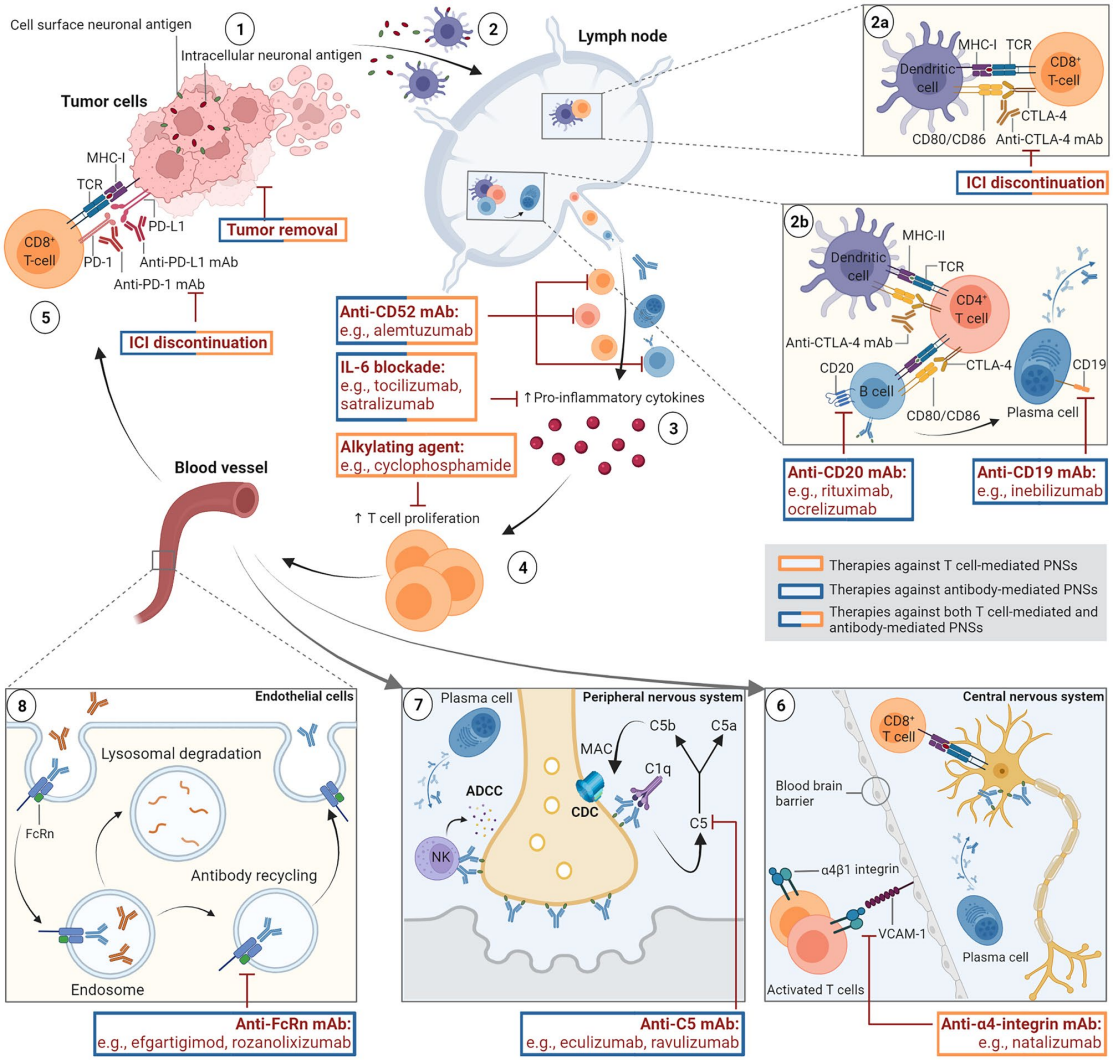
Proposed pathogenic mechanisms of immune checkpoint-inhibitor (ICI)-induced paraneoplastic neurological syndromes (PNSs) and therapeutic strategies. The induction of immune-mediated PNSs under ICI therapy is a multistep process, resulting in the accumulation and amplification of autoreactive cellular and humoral immune responses directed against the central nervous system (CNS) and peripheral nervous system. Tumor neoantigens are released upon tumor necrosis (1). Dendritic cells capture, process and present these cancer-derived neoantigens on major histocompatibility complex (MHC) molecules to naïve T cells in the lymph nodes (2). Recognition of intracellular neuronal antigens (red ovoid shapes) activates CD8^+^ cytotoxic T cells, giving rise to T cell-mediated PNSs (2a). Cell surface neuronal antigens (green ovoid shapes) are recognized by CD4^+^ T helper cells that then activate memory B cells and antibody-producing plasma cells, driving antibody-mediated PNSs (2b). ICIs are monoclonal antibodies (mAbs) that block co-inhibitory signals of T cell activation, including the cytotoxic T lymphocyte antigen 4 (CTLA-4) (2a + b), programmed cell death 1 (PD-1) or its ligand PD-L1 (5), resulting in enhanced T cell activation and proliferation. In addition to cellular changes, ICIs increase the production of pro-inflammatory cytokines (3), which can further promote T cell proliferation (4). Tumor-infiltrating effector CD8^+^ T cells recognize the cognate antigen, which is presented by MHC I molecules on tumor cells, leading to tumor cell killing (5). If CD8^+^ cytotoxic T cells and antibody-secreting plasma cells travel to the nervous system, they can induce PNSs manifesting as immune-related adverse events (irAEs) under ICI therapy. Autoreactive cytotoxic CD8^+^ T cells can cross the blood–brain barrier (BBB) and cause direct cytotoxicity and irreversible neuronal cell death in the CNS (6). Autoantibodies targeting cell surface neuronal antigens can cause cell damage via modulation of protein expression and function, antibody-dependent cellular cytotoxicity (ADCC), or complement-dependent cytotoxicity (CDC) in the CNS (6) or the peripheral nervous system (7). Endothelial cells can recycle immunoglobulin G (IgG) autoantibodies with the neonatal Fc receptor (FcRn) that prevents lysosomal degradation upon binding to the antibody, further contributing to antibody-mediated neuronal damage (8). Therapeutic strategies (red) are guided by the pathogenesis of the PNS and aim at reducing disease-driving autoreactive cytotoxic T cells (orange boxes) or autoantibodies (blue boxes) (see main text for details). Some therapeutic options reduce both pathogenic T cells and autoantibodies (mixed orange and blue boxes). ADCC, antibody-dependent cellular cytotoxicity; APC, antigen-presenting cell; C, complement component; CD, cluster of differentiation; CDC, complement-dependent cytotoxicity; CTLA-4, cytotoxic T lymphocyte-associated antigen; FcRn, neonatal Fc receptor; ICI, immune checkpoint inhibitor, IL, interleukin; mAb, monoclonal antibody; MAC, membrane attack complex; MHC, major histocompatibility complex; NK, natural killer cell; PD-1, programmed cell death protein 1 receptor; PD-L1, programmed cell death protein ligand 1; TCR, T cell receptor VCAM-1, vascular cell adhesion molecule-1. Created with BioRender.com

PNSs are remote complications of systemic cancer that can affect every aspect of the nervous system, including the central nervous system (CNS), the peripheral nervous system, and the neuromuscular junction. These disorders are not directly attributable to the local effects and metastases of the underlying malignancy or indirectly caused by metabolic disturbances, coagulopathies, infections, or treatment-related side effects [15]. Instead, they commonly arise from a cross-reactive autoimmune response against shared autoantigens between cancer cells and the neuronal tissue (Fig. 1) [18]. In a first step, ectopically expressed intracellular or cell surface neuronal antigens, including neoantigens resulting from genetic alterations in cancers cells, are released from necrotic tumor cells (Fig. 1(1)). Antigen-presenting cells (APCs), such as dendritic cells, take up, process, and present these cancer-derived antigens on their surface via major histocompatibility complex class (MHC) I or MHC II molecules to naïve T cells in the lymph nodes, leading to priming and activation of T cells (Fig. 1(2)). Previously primed and activated tumor-infiltrating CD8^+^ cytotoxic T cells bind with their T cell receptor (TCR) to the cognate antigen, which is presented on the surface of cancer cells via MHC I molecules (Fig. 1(5)) [19]. Upon binding to the target antigen, effector T cells generate an antitumor immune response, culminating in tumor cell death.

At times, this immune response is misdirected and gives rise to the production of CD8^+^ cytotoxic T cells and autoantibodies that target the own nervous system (Fig. 1(6) + (7)) [19]. T cell-mediated PNSs are driven by intracellular antigens in the context of TCR binding (Fig. 1(2a)). Antibody-mediated PNSs derive from cell surface antigens that are presented by MHC II molecules on dendritic cells and recognized by CD4^+^ T helper cells. Interactions between activated CD4^+^ T helper cells and B cells result in the generation of memory B cells and antibody-secreting plasma cells (Fig. 1(2b)).

While many tumor cells are known to express neuronal antigens, only a minority of patients with such cancers develop PNSs [17]. Several studies suggest that genetic alterations during oncogenesis, resulting in the expression of highly immunogenic neoantigens by tumor cells, serve as the inciting event to trigger autoimmunity [20, 21]. In a study addressing the genetic characterization of ovarian carcinomas associated with paraneoplastic cerebellar degeneration and anti-Yo antibodies (Yo-PCD), somatic mutations in the Yo antigens CDR2 and CDR2L were found in 65% of the tumors. In 59% of Yo-PCD patients, gene amplification of the CDRL2 gene, encoding for the respective oncoprotein, was detected [20].

Further, anticancer immunity is not always necessarily accompanied by autoimmune neurological toxicity. In an experimental study, transgenic mice expressed the intracellular antigen β-galactosidase in both neurons and implanted tumor cells [22]. In this model, transfer of antigen-specific T cells caused CD8^+^ T cell-mediated tumor lysis without provoking autoimmune neurotoxicity [22].

## ICIs—Boosters of Anticancer Immunity

T cell activation is tightly regulated by counterbalancing co-stimulatory and co-inhibitory signals [23, 24]. The physiologic co-inhibitory immune checkpoints CTLA-4 and PD-1 and its ligand PD-L1 are essential for maintaining immune self-tolerance [23, 24]. CTLA-4 is expressed on activated CD4^+^ T helper cells, CD8^+^ cytotoxic T cells, and regulatory T cells. Binding of CTLA-4 to the ligands CD80 and CD86, which are found on professional APCs, inhibits T cell priming in the lymph nodes, and therefore intercepts T cell activation at an early step (Fig. 1(2a)) [23]. PD-1 is found on the surface of activated T cells, especially tumor-infiltrating T cells, B cells, macrophages, and natural killer cells, and binds to its ligand PD-L1, expressed on APCs and various tumor cells (Fig. 1(5)) [25]. Activation of the CTLA-4 and PD-1/PD-L1 pathways triggers a negative feedback mechanism to inhibit T cell functions which, as a consequence, prevents autoimmunity [23, 24].

Tumor cells can exploit and potentiate the CTLA-4 and PD-1/PD-L1 pathways leading to T cell anergy and T cell apoptosis. The circumvention of T cell-mediated antitumor immunity enables unchecked cancer proliferation [26]. Thus, augmenting the endogenous ability of T cells to mediate antitumor immune responses, resulting in tumor cell killing, has become a major focus in cancer therapeutics [19]. The manipulation of antigen-specific T cell responses to overcome the evasive mechanisms of cancer cells has led to the development of ICIs [27]. The armamentarium of ICIs consists of monoclonal antibodies targeting the co-inhibitory molecules CTLA-4 (e.g., ipilimumab), PD-1 (e.g., nivolumab), and PD-L1 (e.g., atezolizumab) [4]. The therapeutic blockade of the CTLA-4 and PD-1/PD-L1 pathways promotes the activation of tumor-specific T cells resulting in tumor rejection. On the downside, the breakdown of immune tolerance is an undesired outcome of the therapeutic enhancement of T cell activity, manifesting as irAEs.

## ICI-Induced Immune Tolerance Breakdown in PNSs

The exact pathomechanisms by which ICIs induce the diversity of irAEs are not fully elucidated [28]. In the case of PNSs, molecular mimicry due to sequence similarities between neuronal antigens and pathogen-derived antigens resulting in autoreactive T cells is proposed to be a driving pathogenic mechanism. Together with the ICI-induced augmentation of the immune system, the emergence of PNSs in patients with systemic cancer can be promoted. Findings in mouse models regarding the induction of paraneoplastic cerebellar degeneration upon ICI treatment support this hypothesis [29]. In this model, mice expressed a *neo*-self-antigen, which is shared by Purkinje cells and implanted breast tumor cells. Injection of antigen-specific lymphocytes limited tumor growth without causing neurological autoimmunity. Co-administration of an anti-CTLA-4 antibody increased antitumor immunity, however at the cost of neuroinflammation with histopathologic findings of CD8^+^ T cell-mediated Purkinje cell loss in the cerebellum and the consecutive development of paraneoplastic cerebellar degeneration [29].

Another possible pathogenic mechanism of neurologic irAEs is the modulation of humoral immunity with increased B cell-mediated antibody production [30]. This hypothesis is supported by findings in a mouse model, in which repeated injections of anti-CTLA-4 antibodies into mice induced pituitary antibodies [31]. Another study showed that patients with anti-CTLA-4-mediated hypophysitis developed pituitary antibodies that could have contributed to toxicity of anti-CTLA-4 therapy [32]. In addition to misdirected humoral immunity, autoreactive T cells may play an important role in ICI-mediated hypophysitis as endogenous pituitary cells were found to express CTLA-4 antigens [31].

## General Characteristics of PNSs

While the clinical phenotypic presentations of PNSs are heterogeneous, these neurological disorders share some common features. PNSs are rare disorders, presenting in less than 1.0% of cancer patients, but clinically relevant because in up to 65% of patients they predate the diagnosis of an occult, often early-stage malignancy [17, 33]. Therefore, PNSs are often the first sign of a neoplasm. The most commonly recognized tumors in PNSs, accounting for about 73% of cases, include SCLC, breast cancer, ovarian cancer, NSCLC, and lymphoma [33]. The specific type and frequency of an underlying cancer depends on the neurological phenotype of the PNSs, demographic characteristics (age, presence of risk factors, e.g., smoking), and neuronal autoantibody type.

Certain neurological syndromes are more frequently associated with a paraneoplastic etiology and termed high-risk neurological phenotypes [15]. High-risk neurological phenotypes include the following diagnoses: encephalomyelitis, limbic encephalitis, rapidly progressive cerebellar syndrome, opsoclonus myoclonus syndrome (OMS), subacute sensory neuronopathy, gastrointestinal pseudo-obstruction, and Lambert-Eaton myasthenic syndrome (LEMS) [15]. Among these, rapidly progressive cerebellar syndrome and subacute sensory neuronopathy are the most common types of PNSs [33]. Clinical onset of PNSs is usually acute to subacute with a rapidly progressive course that can lead to disabling permanent neurological damage, or even death with a fatality rate of up to 27% [33]. Recognizing and identifying these disorders and their associated cancer allow timely initiation of oncologic treatment to prevent long-term neurological disability or death in patients with the potential of a good clinical outcome due to their limited stage disease [17]. A brief overview of the characteristic clinical presentation, cancer, and antibody association of high-risk PNSs phenotypes is summarized in Table 1.

**Table 1:** Clinical characteristics, antibody and cancer association of high-risk PNSs

**Syndrome**	**Clinical symptoms**	**Antibody association**	**Most common cancer association**	**References**
Encephalomyelitis	Neurological dysfunction involving multiple levels of the nervous system, including the CNS, the peripheral and autonomic nervous system	Hu (ANNA-1) CRMP5 (CV2) MAP1B (PCA-2)	SCLC SCLC SCLC, NSCLC, breast cancer	[34–36]
Limbic encephalitis	Short-term memory deficits, insomnia, behavioral changes, psychosis, seizures	Hu (ANNA-1) Ma2 AMPAR GABA_B_R mGluR5	SCLC Testicular cancer, NSCLC SCLC, thymoma SCLC Hodgkin lymphoma	[37–43]
Rapidly progressive cerebellar syndrome	Ataxia, diplopia, dysarthria, nystagmus	Hu (ANNA-1) Zic4 Yo (PCA-1) Tr (DNER)	SCLC SCLC Ovarian and breast cancer Hodgkin lymphoma	[44–47]
OMS	Involuntary, arrhythmic, multidirectional chaotic saccadic eye movements, myoclonus, cerebellar syndrome, encephalopathy	Ri (ANNA-2) (adults) Glycine receptor Seronegative (children)	Breast cancer Lung cancer Neuroblastoma	[48, 49]
Sensory neuronopathy	Asymmetric hypesthesia, pain, proprioceptive loss, typically involving the arms, motor deficits possible	Hu (ANNA-1) CRMP5 (CV2) Amphiphysin	SCLC SCLC SCLC, breast cancer	[50–52]
Gastrointestinal pseudo-obstruction	Abdominal pain, distension, constipation, nausea, vomiting, dysphagia	Hu (ANNA-1)	SCLC	[53]
LEMS	Proximal muscle weakness starting in the limbs, progressing to involve the upper extremity, facial and ocular muscles, autonomic dysfunction	SOX1 (AGNA-1) P/Q-type VGCC	SCLC SCLC	[54, 55]

*AGNA-1* anti-glial nuclear antibody type 1, *AMPAR* α-amino-3-hydroxy-5-methyl-4-isoxazolepropionic acid receptor, *ANNA* antineuronal nuclear antibody, *CNS* central nervous system, *CRMP5* collapsin response mediator protein 5, *CSF* cerebrospinal fluid, *GABA*_*B*_*R* GABA type B receptor, *DNER* delta and notch-like epidermal growth factor-related receptor, *LEMS* Lambert-Eaton myasthenic syndrome, *MAP1B* microtubule-associated protein 1B, *mGluR5* metabotropic glutamate receptor 5, *NSCLC* non-small-cell lung cancer, *OMS* opsoclonus myoclonus syndrome, *PNS* *p*araneoplastic neurological syndrome, *P/Q-type VGCC* voltage-gated calcium channel, *SCLC* small-cell lung cancer, *SOX1* SRY-like HMG box 1

## Autoantibodies as Biomarkers in PNSs

A mainstay in diagnosing PNSs is the detection of neuronal autoantibodies in the serum or cerebrospinal fluid (CSF) that are detectable in 82% of patients with PNSs [33]. These autoantibodies support an immune-mediated pathogenesis and can guide cancer screening measures as each type of detected antibody occurs with only a few tumor types.

These autoantibodies are usually associated with highly distinct and stereotypical neurological syndromes and can often reliably predict the paraneoplastic nature of the disorder and thus serve as important biomarkers for paraneoplastic autoimmunity [15]. Neuronal autoantibodies can be categorized into two main groups. Autoantibodies targeting intracellular nuclear or cytoplasmic neuronal proteins, previously referred to as onconeuronal antibodies, are highly indicative of an underlying malignancy and classified as high-risk antibodies for their frequent cancer association [15]. High-risk antibodies include Hu (also known as antineuronal nuclear antibody type 1 (ANNA-1)), Yo (also known as Purkinje cell cytoplasmic antibody type 1 (PCA-1)), microtubule-associated protein 1B (MAP1B, also known as Purkinje cell cytoplasmic antibody type 2 (PCA-2)), collapsing response mediator protein 5 (CRMP5, also known as CV2), Ri (also known as antineuronal nuclear antibody type 2 (ANNA-2)), Ma2, amphiphysin, SRY-Box 1 (SOX1, also known as anti-glial nuclear antibody (AGNA-1)), Tr (also known as delta/notch-like epidermal growth factor-related receptor (DNER)), and Kelch-like protein 11 (KLHL11). Glutamate decarboxylase 65 (GAD65) antibodies are an exception to this rule as they are usually not paraneoplastic in origin despite directed against an intracellular antigen [56]. Previous studies have identified Hu and Yo as the most prevalent antibodies in PNSs with a frequency of 39% and 13%, respectively [33, 39]. Experimental studies found that autoantibodies directed against intracellular antigens are not directly pathogenic, because their target proteins are inaccessible to direct antibody binding [57]. Instead, postmortem studies in patients with PNSs and high-risk paraneoplastic antibodies suggest that neurodegeneration is mediated by T cell toxicity, based on the detection of granzyme-B^+^ cytotoxic T cells in close proximity to neurons [58].

Autoantibodies targeting neuronal cell surface proteins have an intermediate risk for tumor association and can occur in the presence or absence of cancer [15]. Hence, the detection of these antibodies does not necessarily indicate a paraneoplastic origin. Autoantibodies with an intermediate risk for cancer association comprise N-methyl-d-aspartate receptor (NMDAR), α-amino-3-hydroxy-5-methyl-4-isoxazolepropionic acid receptor (AMPAR), GABA type B receptor (GABA_B_R), metabotropic glutamate receptor 5 (mGluR5), contactin-associated protein-like 2 (CASPR2), and P/Q-type voltage-gated calcium channel (P/Q-type VGCC) [15]. These antibodies harbor the potential to directly unfold pathogenicity by binding to the target protein, resulting in the modulation of protein expression and function. As the target proteins are often ion channels, antibodies have been found to cause electrophysiologic changes, disturbances in synaptic transmission, and neuronal plasticity [59].

In general, patients with antibodies targeting intracellular neuronal structures are poorly responsive to immunosuppressive therapy. In these patients, histopathologic evidence demonstrates cytotoxic T cell-mediated neurodegeneration with severe neuronal cell loss and axonal dystrophy, which might explain the poor response of these disorders to immunomodulation [58]. Exceptions to this rule apply and include the recently identified anti-glial fibrillar acidic protein (GFAP)-associated meningoencephalomyelitis, which is frequently responsive to immunosuppression, even though GFAP targets intracellular proteins [60]. By contrast, the majority of patients with antibodies binding to surface-expressed CNS antigens shows profound neurologic improvement following immunosuppressive treatment aimed to remove the directly pathogenic antibodies or antibody-producing cells [61–63].

## Autoantibody Profiles in ICI-Mediated PNSs

Neuronal autoantibodies have been detected in 54.0% of patients with ICI-mediated neurological autoimmunity [12]. Most of these autoantibodies had intracellular antigenic specificity, including Ma2, GFAP, Hu, Ri, GAD65, SOX1, and CRMP5, which highlights their role as biomarkers of paraneoplastic neurologic autoimmunity [7, 12, 14, 16, 64]. Neurological dysfunction induced by autoantibodies targeting cell surface molecules, including CASPR2, NMDAR, LGI1, and P/Q-type VGCC, was also described, although less frequently [12, 64–66]. Recently discovered autoantibodies against the intracellular neuronal intermediate filament (NIF) or protein phosphodiesterase 10A (PDE10A) have been identified in the context of ICI treatment. NIF antibodies occurred in patients presenting with cerebellar ataxia and encephalopathy following anti-PD-1 therapy [67]. Patients harboring PDE10A antibodies developed hyperkinetic movement disorders as a paraneoplastic phenomenon after the onset of ICI therapy [68]. Novel autoantibodies, including those with unknown molecular specificity, discovered in neurological irAEs, are not detectable with commercially available antigen-specific cell-based assays. Alternatively, an unbiased neuronal autoantibody testing methodology is preferred. In particular, indirect immunofluorescence on rodent brain tissue, known as tissue-based assay, can assess for the presence of novel autoantibodies [69]. As neuronal autoantibodies are frequently encountered in ICI-mediated neurological autoimmunity, autoantibody testing is recommended to assist in diagnosing these disorders and guiding therapeutic decisions for a favorable patient outcome.

## Clinical Characteristics of PNSs in the Setting of ICI Treatment

Some neurologic irAEs fulfill the criteria for a PNS with the typical (i) clinical syndromic manifestation, (ii) tumor association, and (iii) antibody association [15]. In previous studies, most patients with ICI-mediated PNSs and detectable neuronal autoantibodies had CNS involvement and commonly presented with the stereotypical syndromic manifestation associated with the neuronal antibody [13, 14]. This observation suggests that ICIs may be able to unleash PNSs.

While in spontaneous PNSs neurologic symptoms typically precede the diagnosis of an early-stage cancer, ICI-mediated PNSs develop shortly after the initiation of cancer immunotherapy in patients that were previously diagnosed with an advanced stage malignancy [33, 70]. ICIs can also induce PNSs in non-neuroendocrine malignancies that are not typically linked to paraneoplastic conditions, such as melanoma, non-SCLC, or renal cell carcinoma [12]. Cancer immunoediting resulting in the expression of altered neoantigens has been observed in patients with melanoma when subjected to ICI therapy and might be contributing to increased immunogenicity in cancers not classically recognized for paraneoplastic autoimmunity [71, 72].

Potentially life-threatening PNSs include paraneoplastic encephalitis or myasthenic syndromes [10, 73]. Especially cases with anti-Ma2-associated encephalitis presented with severe neurologic deterioration irresponsive to immunosuppressive therapies and were often fatal [16, 73]. In ICI-related myasthenic syndromes, mortality rates are estimated at 30.4% [74]. The risk for myasthenic crisis and comorbid myositis and myocarditis is higher compared to idiopathic myasthenia gravis [10]. Acetylcholine receptor (AChR) antibodies were detected in about 70% of ICI-associated myasthenic syndromes [10].

Caution is warranted in patients with pre-existent evidence of seropositive PNSs. ICIs can worsen pre-existent PNSs resulting in potentially irreversible neurological dysfunction or even death [12, 73]. Notably, patients with a prior history of paraneoplastic encephalitis or rapidly progressive cerebellar syndrome with seropositivity for neuronal autoantibodies, such as P/Q-type VGCG, amphiphysin, ANNA-1, and CRMP5, presented with severe progression of their neurological disorder without improvement following immunosuppression [12, 73].

This finding underscores the importance of a careful risk–benefit evaluation before initiating ICIs in patients at high risk for severe neurotoxicity. Determining a baseline serologic profile of patients with typical paraneoplastic cancers may aid in predicting the risk for adverse events and guide therapeutic decisions to improve patient outcome. However, prospective studies need to evaluate whether patients with cancers known for their paraneoplastic association and detectable neuronal autoantibodies are at higher risk for developing PNSs in the scenario of immunotherapy.

In the following section, we provide a brief overview of the clinical features associated with ICI-induced PNSs. A full review of the clinical characteristics of PNSs is beyond the scope of this review and has been already reported in detail previously [18].

### Encephalomyelitis

Paraneoplastic encephalomyelitis is characterized by multifocal involvement of the nervous system, including the CNS, the peripheral nervous system, and the autonomic nervous system. The predominant cancer is SCLC and the most commonly detected autoantibodies are Hu and CRMP5 [34, 35]. The diagnosis of paraneoplastic encephalomyelitis is challenging due to multifocal neurological signs. Brain and spine MRI play a pivotal role to evaluate diffuse brain lesions that indicate disseminated encephalomyelitis. Electrodiagnostic studies can show peripheral nerve involvement. Most patients with anti-Hu-associated encephalomyelitis progress despite immunotherapy with or without concurrent antineoplastic treatment leading to neurological disability or death [34].

Encephalomyelitis linked to ICI treatment has been rarely described. Worsening of preexisting anti-Hu- and anti-CRMP5-associated encephalomyelitis following nivolumab therapy for SCLC has been observed [12]. Neurologic outcome was poor even after ICI discontinuation, intravenous (IV) methylprednisolone, intravenous immunoglobulins (IVIGs), plasmapheresis, and cyclophosphamide. This case exemplarily demonstrates the risk of severe neurologic progression in patients with pre-existent seropositive PNSs and urges caution for applying ICIs in this setting.

### Limbic Encephalitis and Beyond

Limbic encephalitis is one of the most common PNSs affecting the CNS. Both paraneoplastic and non-paraneoplastic autoimmune forms of limbic encephalitis exist with similar clinical presentations [75]. The likelihood of cancer association depends on the detected neuronal autoantibody, and the spectrum of associated autoantibodies in limbic encephalitis is wide. While Hu, Ma2, AMPAR, and GABA_B_R autoantibodies are frequently associated with cancers, CASPR2 and LGI1 usually manifest as non-paraneoplastic limbic encephalitis [37, 38, 76]. Typical underlying malignancies are SCLC, testicular germ cell cancer, and Hodgkin lymphoma [39]. A hallmark of clinical presentation is short-term memory dysfunction. Patients further present with insomnia, behavioral changes, psychiatric symptoms, and sometimes seizures [39]. In anti-Ma2-associated encephalitis, additional diencephalic involvement has been observed, clinically manifesting as weight gain, narcolepsy, and hyperphagia [16]. MRI T2/FLAIR hyperintensities in the uni- or bilateral mesiotemporal lobes further suggest limbic system involvement [77]. CSF analysis typically reveals inflammatory changes with lymphocytic pleocytosis [39].

Paraneoplastic limbic encephalitis related to ICI treatment has been observed in patients with SCLC, but also in tumors not typically associated with PNSs, including melanoma and myxoid chondrosarcoma [18, 65, 78–81]. Neuronal autoantibodies including CASPR2, GAD65, Ma2, Hu, SOX1, and P/Q-type VGCC, as well as seronegative cases, have been reported in ICI-mediated limbic encephalitis [7, 65, 78, 80, 82, 83]. Except for anti-SOX1 and P/Q-type VGCC antibodies, which are usually associated with LEMS, the remaining autoantibodies resemble those that are frequently present in spontaneous paraneoplastic and autoimmune limbic encephalitis. Overall, most cases of ICI-induced limbic encephalitis demonstrated marked neurological improvement or resolution with ICI discontinuation and steroids as first-line therapy, except for anti-Ma2 positive cases that are associated with a poor neurologic outcome [64]. For worsening symptoms, second-line therapy with IVIGs or rituximab can be beneficial [12, 78, 79]. Natalizumab has also been successfully used in this setting [83].

Autoimmune encephalitis related to NMDAR autoantibodies is considered an intermediate-risk paraneoplastic phenotype as cancer association depends on sex and age. Anti-NMDAR encephalitis presents with prominent psychiatric symptoms, memory deficits, orofacial dyskinesia, seizures, and rapid deterioration of level of consciousness, at times requiring ventilatory support. These symptoms often develop shortly after a prodromal episode with viral-like illness [84]. The presentation of these symptoms in young women should promptly warrant cancer screening for ovarian teratoma, as up to 60% of women harvest these tumors [85]. Only a few cases of anti-NMDAR encephalitis have been reported in the context of ICI therapy in patients with cancers not typically associated with these PNSs, including melanoma, Merkel cell carcinoma, and endometrial adenocarcinoma [12, 78, 86]. Previous studies have revealed that malignant melanomas harbor somatic mutations in the gene *GRIN2A* which encodes for the NMDAR subunit NR2A, resulting in loss of NMDAR complex formation, suggesting that altered expression of neuronal antigens in melanoma cells in conjunction with immune stimulation with ICIs might have triggered the production of pathogenic autoantibodies [87, 88].

The lack of patients with ovarian carcinoma might be explained by the fact that ICIs are currently not approved for the treatment of ovarian cancer. Previous studies have observed neurologic improvement in two cases of ICI-mediated anti-NMDAR encephalitis following rituximab as first- or second-line therapy [12, 78], whereas IV methylprednisolone and plasmapheresis were ineffective [86].

### Rapidly Progressive Cerebellar Syndrome

Rapidly progressive cerebellar syndrome manifests with rapidly evolving ataxia, diplopia, dysarthria, and nystagmus [89–91]. Paraneoplastic cerebellar dysfunction is mostly associated with SCLC and Hu or Zic4 antibodies, gynecological and breast cancer with Yo antibodies, and Hodgkin lymphoma with DNER or mGluR1 antibodies [92]. MRI abnormalities are absent in most patients. In some cases, mild cerebellar atrophy, cerebellar edema, and diffuse T2/FLAIR hyperintensities in the cerebellar cortex have been observed [12, 64, 89]. CSF abnormalities comprise inflammatory changes with mild lymphocytic pleocytosis, protein elevation, and in some cases oligoclonal bands [64].

In the context of ICI treatment, rapidly progressive cerebellar syndrome developed in patients with underlying NSCLC, SCLC, bladder cancer, Hodgkin lymphoma, and melanoma [64, 89–91]. Neuronal autoantibodies including Hu, NIF, CRMP5, amphiphysin, and P/Q-type VGCC, as well as antibodies with atypical neuropil staining, along with seronegative cases, have been reported in this setting [64, 89–91]. Marked clinical improvement was observed especially in seronegative cases of rapidly progressive cerebellar syndrome following ICI withdrawal and steroid therapy. However, anti-Hu-associated cerebellar dysfunction and additional administration of IVIG only provided moderate resolution [12, 64].

### Opsoclonus Myoclonus Syndrome

OMS is characterized by involuntary, arrhythmic, multidirectional chaotic saccadic eye movements and is commonly accompanied by myoclonus and a cerebellar syndrome (ataxia, dysarthria) [48, 93]. In children, OMS is strongly associated with the presence of neuroblastoma, whereas in adults the most common underlying tumors are breast cancer associated with Ri antibodies and lung cancer associated with glycine receptor antibodies [49]. MRI and CSF findings are often unremarkable. To date, one case of OMS has been reported in a patient with mesothelioma following anti-CTLA-4 and anti-PD-1 combination therapy [94]. Neurologic symptoms resolved with high-dose methylprednisolone along with IVIGs.

### Sensory Neuronopathy

Sensory neuronopathy (SN) presents with subacute onset of asymmetric hypesthesia, severe pain, and loss of proprioception, typically involving the arms, and sometimes occurs with motor deficits [51]. Electrodiagnostic studies reveal involvement of the dorsal root ganglia of sensory neurons. These syndromes are frequently associated with SCLC and Hu or CRMP5 antibodies [51]. SN has been reported in two patients with melanoma and SCLC following ICI therapy [13, 95]. In the latter, anti-Hu antibodies were detected. In both patients, neurological symptoms improved following ICI discontinuation and steroid therapy.

### Lambert-Eaton Myasthenic Syndrome

LEMS is a neuromuscular junction disorder presenting with proximal muscle weakness starting in the limbs, progressing to involve the upper extremity, facial, and ocular muscles. Progressive muscle weakness is often accompanied by autonomic dysfunction and generalized fatigue. Pathogenic antibodies directed against the presynaptic P/Q-type VGCC are detected in up to 90% of patients with either paraneoplastic or non-paraneoplastic LEMS, while SOX1 antibodies are highly indicative of an underlying SCLC [54, 55]. Repetitive nerve stimulation demonstrating an increase in the compound muscle action potential (increment) is supportive of LEMS. While myasthenic syndromes have been increasingly recognized as severe manifestations of neurologic irAEs, occurring in about 0.1–0.2% of patients receiving ICIs, only a few cases of ICI-related LEMS have been described [66, 96–98]. All cases were positive for P/Q-type VGCC autoantibodies and the underlying tumors were SCLC and NSCLC. Marked clinical improvement was only achieved in one case with IVIGs and 3,4-diaminopyrimidine [98], while in the two remaining patients rituximab as second-line therapy [97] and steroids as first-line therapy did not resolve neurological disability [66].

## Diagnostic Approach

Recognizing PNSs related to ICI therapy is often challenging, given their rarity and broad differential diagnoses. Important alternative causes include infections, metabolic disorders, tumor progression, or complications due to other therapeutic modalities.

New onset of acute or subacute neurologic symptoms in patients recently commencing ICI treatment raises suspicion for a neurologic irAE. All patients should undergo a thorough neurologic evaluation to localize the region of involvement. In patients with suspected paraneoplastic CNS disorder, MRI of the brain and spine can provide imaging evidence to the underlying pathology and rule out metastatic disease of the brain or leptomeninges. CSF analysis should include infectious studies to exclude bacterial, viral, and fungal meningitis and cytopathology to search for clues of leptomeningeal carcinomatosis [99]. Testing for neuronal autoantibodies in the CSF and serum is recommended, as they are frequently positive in irAEs affecting the CNS [12]. In patients with suspected peripheral nervous system involvement, electrodiagnostic findings from electromyography and nerve conduction studies can indicate an immune-related neuropathy, such as non-length-dependent polyradiculoneuropathy or cranial neuropathy [95]. Immune-related neuropathies have to be distinguished from toxic neuropathies related to chemotherapy, which commonly present as symmetric length-dependent sensory polyneuropathy. In addition, neuronal autoantibody panels in the CSF and serum may reveal paraneoplastic antibodies, such as Hu or CASPR2.

For suspected neuromuscular disorders, including myasthenia gravis and LEMS, antibodies against AChR, muscle-specific tyrosine kinase (MuSK), titin, SOX1, and P/Q-type VGCC should be determined. Repetitive nerve stimulation revealing decrement or increment is supportive of myasthenia gravis and LEMS, respectively.

An interdisciplinary team consisting of oncologists and neurologists is required to timely diagnose ICI-related PNSs and initiate appropriate therapeutic measures to substantially reduce patient morbidity and mortality.

## General Management Recommendations of ICI-Induced PNSs

Multinational and multidisciplinary organizations, including the *American Society of Clinical Oncology* (ASCO), the *European Society for Medical Oncology* (ESMO), the *Society for Immunotherapy of Cancer* (SITC), and the *National Comprehensive Cancer Network* (NCCN), have issued comprehensive outlines for the management of irAEs [99–102]. The specific therapeutic guidelines depend on the involved organ system and the severity of the toxicity. The severity of irAEs is graded according to the National Cancer Institute’s Common Terminology Criteria for Adverse Events (CTCAE) into grades 1–5, which in increasing order corresponds to mild (1), moderate (2), severe (3), life-threatening (4), and death due to immunotherapy (5) [103].

Patients with neurologic irAEs should be referred to neurologists with expertise in neuro-oncology. PNSs related to ICI treatment are generally considered grade > 2 neurotoxicities due to the severe clinical course that almost always leads to disabling neurological sequalae. The cornerstones of first-line management are the discontinuation of ICIs and corticosteroid treatment as most patients improve with these therapeutic measures [5, 7, 8, 12]. Current guidelines advise against the reintroduction of ICI therapy in severe neurologic irAEs [99].

Steroids are administered as either oral prednisone (1 mg/kg daily over 4–6 weeks with a slow steroid taper) or as intravenous methylprednisolone (1–4 mg/kg per day or pulse dose with 1000 mg daily for 3–5 days). It has been promising that no clear evidence of systemic steroid use negatively impacting overall survival in oncologic patients with irAEs has been established [104, 105].

In anticipated prolonged and steroid-refractory cases, escalation of immunosuppressive therapy is indicated to prevent permanent neurological dysfunction. IVIGs are currently recommended for Guillain-Barré syndrome, myasthenia gravis, severe or progressive encephalitis, and transverse myelitis following ICI therapy [106]. Several studies have also demonstrated neurological improvement with IVIGs in patients with ICI-mediated PNSs, such as limbic encephalitis [7, 10, 12, 13]. IVIGs are typically administered with standard protocol at 2 g/kg over the course of 5 days. Plasmapheresis with 5–7 cycles every other day has been effective in antibody-mediated neurotoxicities, including myasthenia gravis or autoimmune encephalitis [99].

More aggressive immunosuppressive options may be needed in certain cases and should be guided by the pathogenesis (T cell-mediated versus antibody-mediated) of the PNSs based on the detected autoantibody profile. PNSs associated with antibodies against intracellular antigens are mainly mediated by cytotoxic T cells and rarely improve with treatment. Patients with directly pathogenic neuronal autoantibodies targeting surface antigens commonly respond to immunotherapies that deplete disease-driving antibodies [61–63]. In the following section, we discuss emerging and potential novel immunotherapeutic concepts of ICI-mediated PNSs and focus on therapeutic antibody-selective immunotherapies. Figure 1 depicts therapeutic options according to the proposed mechanisms of ICI-induced PNSs.

### Rituximab

B cell-depleting therapies are proposed to exert their effects by diminishing dysfunctional autoantibody production and by modulating autoreactive pro-inflammatory T cell activity [107, 108]. In particular, anti-CD20 monoclonal antibodies, such as rituximab, have demonstrated to be highly effective in autoimmune neurological disorders [109]. Rituximab is a monoclonal chimeric antibody targeting the transmembrane protein CD20, which is primarily found on the surface of premature and differentiated B cells, but is absent on mature plasma cells (Fig. 1(2b)). Binding of rituximab to the cell surface protein CD20 induces a selective depletion of circulating CD19^+^ and CD20^+^ B cells, except for the terminally differentiated plasma cells [110]. Anecdotal case reports and small case series have reported beneficial effects of second-line rituximab therapy in refractory ICI-related neurotoxicities, including anti-GAD65-associated limbic encephalitis [111], seronegative encephalitis [112], or anti-NMDAR encephalitis [12, 78]. Rituximab was commonly administered at 375 mg/m^2^ weekly over 4 weeks [99]. The absence of detectable autoantibodies is no reason to discard rituximab therapy. Previous studies demonstrated that the efficacy of rituximab was independent from antibody status in patients with autoimmune limbic encephalitis and thus comparable between patients with autoantibodies that were undetectable, directly pathogenic or directed against intracellular antigens [113]. Further CD20-targeting monoclonal antibodies, such as ofatumumab, obinutuzumab, and the humanized equivalent ocrelizumab, might be considered alternatives for rituximab, given their similar efficacy and safety profile, although there is a paucity of evidence for their application in this setting [108].

### Natalizumab

Natalizumab is a humanized monoclonal antibody that selectively targets α_4_-integrin on the surface of lymphocytes, which binds to vascular cell adhesion molecule-1 (VCAM-1) expressed on endothelial cells of the blood–brain barrier (BBB) (Fig. 1(6)). Blocking of α_4_-integrin prevents endothelial transmigration of activated T lymphocytes across the BBB into the CNS [114]. Natalizumab can therefore limit CNS inflammation without affecting systemic immune effects in other compartments. Natalizumab was successfully applied in a patient with ICI-mediated anti-Hu-associated limbic encephalitis, where neuronal damage is suggested to be the result of pathogenic T cell-mediated immunity [58, 83]. This finding supports the use of natalizumab in ICI-mediated paraneoplastic encephalitis as the prevention of pathogenic T cell infiltration across the BBB can block the inflammatory cascade within the CNS to start.

### Cyclophosphamide

Active metabolites of cyclophosphamide are alkylating agents that form cross-links within DNA strands leading to profound effects on T cell function and thereby decreasing the immune response (Fig. 1(4)) [115]. As cyclophosphamide preferentially targets T lymphocytes, it is often used in PNSs mediated by a cytotoxic T cell response [116, 117]. Improvement in neurologic symptoms following cyclophosphamide as second-line therapy has been observed in ICI-mediated PNSs with a classic paraneoplastic association, including anti-Hu and anti-CRMP5 antibody-positive cerebellar ataxia with peripheral neuropathy and encephalomyelitis, anti-Hu and striational antibody-positive limbic encephalitis with cerebellar ataxia and cranial neuropathy, and anti-CRMP5-positive progressive myelopathy [12, 118].

## Potential Future Biologics Therapies

### Anti-CD19 Therapy

Inebilizumab is a humanized monoclonal antibody targeting the transmembrane protein CD19, which is widely expressed on all B lineage cells, including plasma blasts and plasma cells (Fig. 1(2b)) [119]. Treatment with inebilizumab induces a depletion of CD19^+^ B cells via antibody-dependent cellular cytotoxicity (ADCC) and may target a larger proportion of pathogenic B cells compared to the CD20-depleting agent rituximab [120]. The phase II/III clinical trial N-MOmentum reported reduced relapse rates in NMOSD patients treated with inebilizumab, which is currently approved for the treatment of aquaporin-4 antibody (AQP4)-positive NMOSD in adults [121]. Ongoing clinical trials investigate the use of inebilizumab for the therapy of myasthenia gravis. The results from the N-MOmentum trial suggest that CD19-depleting agents have the potential to provide benefit in antibody-mediated diseases, which may extend into the setting of PNSs, induced by humoral autoimmunity.

### Anti-CD52 Therapy

Alemtuzumab is a humanized monoclonal antibody targeting the glycoprotein CD52, which is predominantly expressed on the surface of mature B and T cells (Fig. 1(2)), but is also found at lower levels on cells of the innate immune system, including eosinophils, monocytes, macrophages, neutrophils, and natural killer cells [122]. Treatment with alemtuzumab induces depletion and reconstitution of circulating CD52^+^ B and T lymphocytes, resulting in sustained changes of the adaptive immunity, which is thought to contribute to the clinical benefits in autoimmune disorders [123]. Alemtuzumab is currently approved for the therapy of relapsing–remitting multiple sclerosis [124]. Individual case reports and small case series describe successful application of alemtuzumab in the setting of seropositive paraneoplastic disorders, including cancer-associated retinopathy (CAR) with detectable CAR autoantibodies [125] and desmoglein 3- and desmoplakin I- and II-positive paraneoplastic pemphigus (PNP) [126]. The immunopathologic mechanisms underlying PNP involve activated autoreactive T cells that promote both humoral and cell-mediated immunity, and in CAR, the majority of autoantibodies are directed against intracellular antigens [127]. These experiences might translate into T cell-mediated paraneoplastic neurological disorders. However, a pitfall of alemtuzumab therapy is the development of secondary antibody-mediated autoimmune disorders (most commonly thyroid disease), which is proposed to be driven by increased interleukin (IL) 21 levels, mediating the expansion of self-antigen-responsive T cells [128, 129].

### Anti-neonatal Fc Receptor Therapy

Novel immunotherapeutic approaches are evolving that aim at reducing pathogenic autoantibodies by inhibiting the neonatal Fc receptor (FcRn) for immunoglobulin G (IgG). The FcRn binds to IgG or albumin and thereby prevents their lysosomal degradation in endothelial cells (Fig. 1(8)). Thus, the FcRn contributes to protective humoral immunity by maintaining IgG and albumin homeostasis [130, 131]. Phase II clinical trials have reported reduced pathogenic anti-AChR IgG concentrations in patients with myasthenia gravis receiving the monoclonal therapeutic antibodies efgartigimod or rozanolixizumab that both target the FcRn [132, 133]. Ongoing phase III clinical studies are assessing the efficacy of these novel therapeutics. Exploratory results suggest that anti-FcRn therapies have the potential to provide clinical benefit in patients with myasthenia gravis. Future therapeutic implications of anti-FcRn may extend beyond de novo autoimmune disorders into the context of PNSs.

### Anti-interleukin-6 Therapy

IL-6 is one of the main cytokines that at high levels can promote inflammatory processes and is involved in the pathogenesis of several autoimmune diseases, including neuromyelitis optica spectrum disorder (NMOSD) [134]. Inhibition of IL-6 signaling with the monoclonal antibodies tocilizumab or satralizumab that target the IL-6 receptor (IL-6R) (Fig. 1(3)) suppresses pro-inflammatory cascades and has shown to ameliorate neurologic symptoms and relapse rates in patients with NMOSD refractory to multiple immunosuppressive therapies [135–137]. Tocilizumab has improved neurological symptoms in a patient with ICI-related steroid-refractory transverse myelitis with high levels of IL-6 in the CSF [138] and demonstrated efficacy in patients with ICI-mediated cerebritis [139]. Satralizumab is a modified anti-IL-6R antibody with a pH-dependent antibody-antigen binding which prolongs the elimination half-life of the drug and has been recently FDA-approved for the treatment of NMOSD [140]. Until now, no cases of ICI-mediated PNSs treated with satralizumab have been reported. Emerging evidence indicates that high IL-6 levels correlate with disease activity and CNS inflammation, while a decrease in IL-6 levels reflects treatment response in NMOSD [135, 141]. Measuring IL-6 levels in the serum or CSF in ICI-mediated CNS inflammation can help to determine patients that might benefit from therapeutic anti-IL-6 blockade.

### Anti-C5 Therapy

The C5 inhibitors eculizumab or ravulizumab are humanized monoclonal antibodies that directly bind to the terminal complement component C5, inhibiting enzymatic cleavage to the fragments C5a and C5b (Fig. 1(7)). As a result, C5a-mediated chemotaxis of inflammatory cells and C5b-mediated formation of the terminal membrane attack complex (MAC) are inhibited, preventing complement-induced inflammation and cytolysis, respectively [142]. Preclinical studies of AChR IgG-positive myasthenia gravis and AQP4-IgG-positive NMOSD have demonstrated complement-mediated neuronal damage, which was absent with the administration of complement inhibitors [143–145]. Based on the results of the REGAIN and PREVENT studies, eculizumab is now FDA-approved for refractory AChR IgG-positive myasthenia gravis and AQP4-IgG-positive relapsing NMOSD [146, 147]. Ravulizumab has been re-engineered from eculizumab to extend its half-life and is currently approved for the treatment of paroxysmal nocturnal hemoglobinuria and atypical hemolytic uremic syndrome [148, 149]. In the current management guidelines, neither eculizumab nor ravulizumab is listed as therapeutic options for neurological irAEs. Future research needs to identify ICI-induced PNSs, in which complement-mediated membrane damage and inflammation plays a pivotal role in the pathogenesis of the disease to expand the use of anti-C5 inhibitors beyond de novo autoimmune neurological disorders.

## Conclusion

Immune checkpoint inhibitors have become a crucial pillar of cancer therapies, yet they can be complicated by life-threatening PNSs that bear the potential for severe and permanent disability. Timely diagnosis and improved treatment algorithms of ICI-triggered PNSs are invaluable for a favorable patient outcome and require knowledge of the diverse spectrum of clinical presentations. A thorough differential work-up is necessary to distinguish these neurotoxic effects from complications of the underlying malignancy or complications of other treatment modalities. Testing for neuronal autoantibodies in the serum and CSF is recommended in suspected cases and, if present, can indicate an enhanced immune-mediated process. Further, the detected autoantibody profile (intracellular versus synaptic antibodies) can guide therapeutic measures. Novel targeted therapeutic approaches with antibody-selective immunotherapies should be implemented in PNSs to avoid serious adverse effects that are commonly seen with established chronic immunosuppressants. In light of the broadened indications for ICIs, especially in cancers strongly associated with PNSs, improved diagnostic and therapeutic algorithms for ICI-triggered PNSs are invaluable to reduce patient morbidity and mortality. Future research is needed to determine biomarkers to identify patients at risk for ICI-induced PNSs.

## Supplementary Information

Below is the link to the electronic supplementary material.

Supplementary file1 (PDF 509 KB)

Supplementary file2 (PDF 460 KB)

## Glossary

AChR: Acetylcholine receptor
ADCC: Antibody-dependent cellular cytotoxicity
AGNA-1: Anti-glial nuclear antibody type 1
AMPAR: α-Amino-3-hydroxy-5-methyl-4-isoxazolepropionic acid receptor
ANNA: Antineuronal nuclear antibody
APC: Antigen-presenting cells
bbb: Blood-brain barrier
CAR: Cancer-associated retinopathy
CASPR2: Contactin-associated protein-like 2
CDC: Complement-dependent cytotoxicity
CNS: Central nervous system
CRMP5: Collapsin response mediator protein 5
CSF: Cerebrospinal fluid
CTCAE: Common Terminology Criteria for Adverse Events
CTLA-4: Cytotoxic T lymphocyte associated antigen 4
DNER: Delta and notch-like epidermal growth factor-related receptor
FcRn: Neonatal Fc receptor
GABA_B_R: GABA type B receptor
GFAP: Glial fibrillar acidic protein
ICI: Immune checkpoint inhibitor
IL: Interleukin
Ig: Immunoglobulin
IrAE: Immune-related adverse event
IV: Intravenous
IVIG: Intravenous immunoglobulin
KLHL11: Kelch-like protein 11
LEMS: Lambert-Eaton myasthenic syndrome
LGI1: Leucine-rich glioma-inactivated protein 1
MAP1B: Microtubule-associated protein 1B
mGluR5: Metabotropic glutamate receptor 5
MHC: Major histocompatibility complex class I/II
MRI: Magnetic resonance imaging
MuSK: Muscle-specific tyrosine kinase
NIF: Neuronal intermediate filament
NK: Natural killer cell
NMDAR: N-methyl-d-aspartate receptor
NMOSD: Neuromyelitis optica spectrum disorder
NSCLC: Non-small-cell lung cancer
GAD65: Glutamate decarboxylase 65
OMS: Opsoclonus myoclonus syndrome
PCA: Purkinje cell cytoplasmic antibody
PCD: Paraneoplastic cerebellar degeneration
PD-1: Programmed cell death protein 1 receptor
PDE10A: Protein phosphodiesterase 10A
PD-L1: Programmed cell death protein ligand 1
PNP: Paraneoplastic pemphigus
PNS: Paraneoplastic neurological syndrome
P/Q-type VGCC: P/Q-type voltage-gated calcium channel
SCLC: Small-cell lung cancer
SOX1: SRY-like HMG box 1
VCAM-1: Vascular cell adhesion molecule-1
